# Improved lipid production via fatty acid biosynthesis and free fatty acid recycling in engineered *Synechocystis* sp. PCC 6803

**DOI:** 10.1186/s13068-018-1349-8

**Published:** 2019-01-04

**Authors:** Kamonchanock Eungrasamee, Rui Miao, Aran Incharoensakdi, Peter Lindblad, Saowarath Jantaro

**Affiliations:** 1 Laboratory of Cyanobacterial Biotechnology, Department of Biochemistry, Faculty of Science, Chulalongkorn University, Bangkok, 10330 Thailand; 2 Microbial Chemistry, Department of Chemistry–Ångström, Uppsala University, Box 523, 75120 Uppsala, Sweden

**Keywords:** Total lipid, Unsaturated lipid, *Synechocystis* sp. PCC 6803, Acyl–acyl carrier protein synthetase, Lipase A, Acyl-ACP reductase, Acetyl-CoA carboxylase

## Abstract

**Background:**

Cyanobacteria are potential sources for third generation biofuels. Their capacity for biofuel production has been widely improved using metabolically engineered strains. In this study, we employed metabolic engineering design with target genes involved in selected processes including the fatty acid synthesis (a cassette of *accD*, *accA*, *accC* and *accB* encoding acetyl-CoA carboxylase, ACC), phospholipid hydrolysis (*lipA* encoding lipase A), alkane synthesis (*aar* encoding acyl-ACP reductase, AAR), and recycling of free fatty acid (FFA) (*aas* encoding acyl–acyl carrier protein synthetase, AAS) in the unicellular cyanobacterium *Synechocystis* sp. PCC 6803.

**Results:**

To enhance lipid production, engineered strains were successfully obtained including an *aas*-overexpressing strain (OX*Aas*), an *aas*-overexpressing strain with *aar* knockout (OX*Aas*/KO*Aar*), and an *accDACB*-overexpressing strain with *lipA* knockout (OX*AccDACB*/KO*LipA*). All engineered strains grew slightly slower than wild-type (WT), as well as with reduced levels of intracellular pigment levels of chlorophyll *a* and carotenoids. A higher lipid content was noted in all the engineered strains compared to WT cells, especially in OX*Aas*, with maximal content and production rate of 34.5% w/DCW and 41.4 mg/L/day, respectively, during growth phase at day 4. The OX*AccDACB*/KO*LipA* strain, with an impediment of phospholipid hydrolysis to FFA, also showed a similarly high content of total lipid of about 32.5% w/DCW but a lower production rate of 31.5 mg/L/day due to a reduced cell growth. The knockout interruptions generated, upon a downstream flow from intermediate fatty acyl-ACP, an induced unsaturated lipid production as observed in OX*Aas*/KO*Aar* and OX*AccDACB*/KO*LipA* strains with 5.4% and 3.1% w/DCW, respectively.

**Conclusions:**

Among the three metabolically engineered *Synechocystis* strains, the OX*Aas* with enhanced free fatty acid recycling had the highest efficiency to increase lipid production.

## Background

**Fig. 1:**
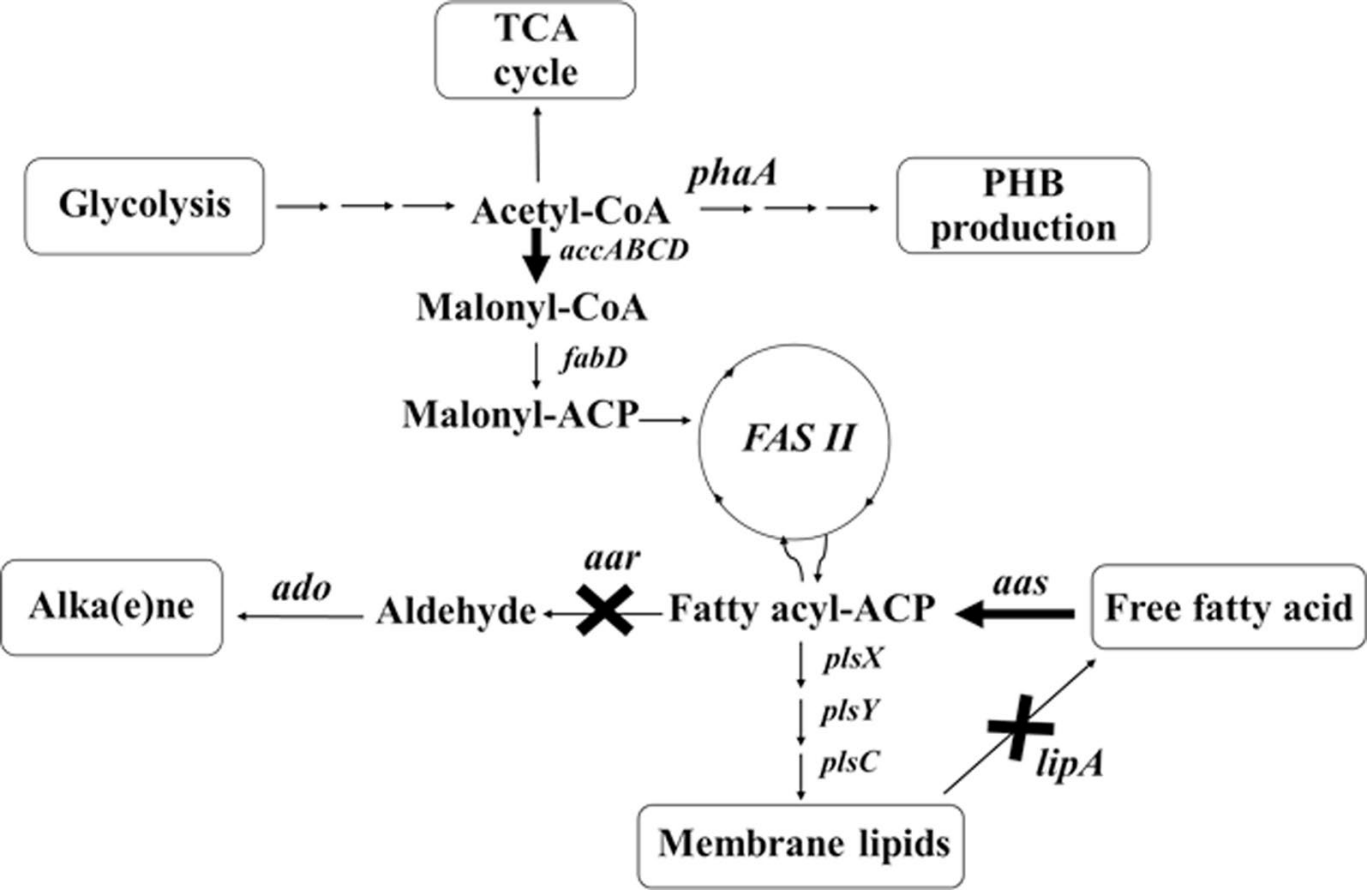
The fatty acid biosynthesis and its neighboring pathways in *Synechocystis* sp. PCC 6803. Key enzyme genes include *accABCD,* multi-subunit acetyl-CoA carboxylase gene; *aar,* acyl-ACP reductase gene; *aas,* acyl-ACP synthetase; *ado,* aldehyde oxidase; *fabD,* malonyl coenzyme A-acyl carrier protein transacylase; *lipA*, lipolytic enzyme genes; *plsX, plsY, plsC,* putative phosphate acyl-transferases; *phaA*, polyhydroxyalkanoates specific beta-ketothiolase gene. The thick arrow is represented as the overexpression (OX) of that gene whereas the cross symbol is represented the knockout (KO) of that gene

Cyanobacteria have recently been used as the third-generation biofuel resources [1] due to their availability of various valuable precursors such as lipids, alkenes, alkanes, PHB and fatty alcohols for biofuel and biodiesel syntheses [2–4]. In addition, they possess a prominent photosynthetic machinery and minimal utilization of basic nutritional requirement with further converting and recycling CO_2_ into fuels and chemicals [1]. The oil productivity of several microalgae greatly exceeds that of oil crops, which allows them to have economic competitiveness with petro-diesel for transportation fuel [5]. Metabolic engineering technology approach and genome sequence databases of cyanobacteria may be used as potential tools for developing cell production competency of energy containing biomolecules or biofuel products. For the lipid synthetic pathway in cyanobacteria (as shown in Fig. 1), the core metabolite acetyl-CoA is converted to fatty acyl–acyl carrier protein (fatty acyl-ACP) via fatty acid synthesis II (FAS II). The first limiting step of lipid biosynthesis begins with acetyl-CoA carboxylase (ACC) catalyzing a carboxylation reaction of acetyl-CoA to malonyl-CoA. In higher plants, the acetyl-CoA pool, which originates from the Calvin cycle and the breakdown of both carbohydrates and lipids, remained relatively unchanged in the range of 30–50 μM except the fatty acid synthesis whose rates varied significantly [6]. Previously, an engineered ACC overexpressing strain of *Escherichia coli* showed a sixfold increased fatty acid level [7]. In the cyanobacterial FAS II system, long-chain acyl-ACP or fatty acyl-ACP is mainly used as a key precursor for phospholipid production [8, 9]. The biochemical balance of fatty acyl-ACP is either gained or reduced as described via neighboring pathways (Fig. 1). The key enzyme for free fatty acid recycling to fatty acyl-ACP in *Synechocystis* is acyl–acyl carrier protein synthetase (AAS) encoded by *aas* which requires ATP, ACP-SH (acyl carrier protein-SH) and cofactors including Mg^2+^ and Ca^2+^ [3, 10]. However, an intermediate flux limitation exists when excess levels of fatty acyl-ACP cause a decreased activity of acetyl-CoA carboxylase (ACC) via a feedback regulation in the fatty acid synthetic processes [11, 12]. An efficient in vivo flow of fatty acyl-ACP intermediate is directed not only to phospholipid production but also, indirectly, to alk(e)ane production [4]. A previous report revealed that overexpression of both *aar/ado*, encoding acyl-ACP reductase and aldehyde dehydrogenase, in alk(e)ane synthetic pathway resulted in an enhanced alk(e)ane production, especially heptadecane, in *Synechococcus* sp. NKBG15041c strain [13]. The direct conversion from fatty acyl-ACP to phospholipids in cyanobacteria has been addressed via a set of PlsX/PlsY/PlsC acyltransferase catalytic systems [14]. The phospholipid homeostasis is maintained via both synthesis and degradation. Recently, the key enzyme for phospholipid hydrolysis to FFA in *Synechocystis* sp. PCC 6803 was identified, a lipase A encoded by *sll1969*, although its regulatory or inducible mechanism remains unclear [15]. Interestingly, many recent reports revealed the competency of modern metabolic engineering to overcome those intracellular-biochemical limitation, in particular feedback inhibitions. For instance, to decrease the costly fatty acid recovery, a so-called damaging cyanobacterial cell membranes strategy was employed, e.g., an acyl-ACP thioesterase overexpression in order to secrete FFA into culture medium [16, 17].

In this study, we generated three metabolically engineered *Synechocystis* 6803 strains: OX*Aas*—*aas*-overexpression, OX*Aas*/KO*Aar*—*aas*-overexpression with *aar* gene interruption, and OX*AccDACB*/KO*LipA*—*accDACB*-overexpression with *lipA* gene interruption (Fig. 1). Our results demonstrate a significant increase of lipid production in all engineered *Synechocystis* 6803 strains.

## Methods

### Strains and growth conditions

*Synechocystis* sp. strain PCC 6803 was grown under normal growth condition of BG_11_ medium at 28 °C under a continuous light illumination intensity of 40 µmol photons/m^2^/s. All engineered strains, OX*Aas*, OX*AccDACB/*KO*LipA* and OX*Aas*/KO*Aar* (Table 1), were grown in a BG_11_ medium containing 35 µg/mL of chloramphenicol. *Escherichia coli* DH5α strain was used as a host propagation and grown at 37 °C on the Luria–Bertani (LB) agar medium containing 30 µg/mL of chloramphenicol. The pre-cultivation was performed initially on BG_11_ agar plates and transferred to 100 mL-liquid medium until cells reaching mid-log phase of growth before starting the experiment. The initial cell density of *Synechocystis* cells for a culture experiment was set at the optical density at 730 nm (OD_730_) of about 0.15. Growth measurement was monitored by a spectrophotometer at OD_730_. Dry cell weight (DCW) was performed by incubating the harvested cells in 60 °C oven until obtaining a constant dry weight.

**Table 1:** Strains and plasmids used in this study

Name	Relevant genotype	Reference
Cyanobacterial strains		
*Synechocystis* sp. PCC 6803	Wild type	Pasteur culture collection
OX*Aas*	*aas, cm*^*r*^ integrated at region of native *aas* gene in *Synechocystis* genome	This study
OX*AccDACB*/KO*LipA*	*accDACB, cm*^*r*^ integrated at flanking region of *lipA* gene in *Synechocystis* genome	This study
OX*Aas*/KO*Aar*	*aas, cm*^*r*^ integrated at flanking region of *aar* gene in *Synechocystis* genome	This study
Plasmids		
pEERM	P_psbA2_-*cm*^*r*^; plasmid containing flanking region of *psbA2* gene	Englund et al. [18]
pEERM_*Aas*	P_psbA2_-*aas*-*cm*^*r*^; integrated between *Xba*I and *Spe*I sites of pEERM	This study
pEERM_*LipA/AccDACB*	P_psbA2_-US*lipA*-*accDACB*-DW*lipA*-cm^r^; integrated between *Xba*I and *Spe*I sites of pEERM	This study
pEERM_*Aar/Aas*	P_psbA2_-US*aar*-*aas*-DW*aar*-cm^r^; integrated between *Xba*I and *Spe*I sites of pEERM	This study

P_psbA2_, strong *psbA2* promoter; *cm*^*r*^, chloramphenicol resistance cassette

### Construction of recombinant plasmids

The pEERM plasmid [18] was used as a cloning and expression vector in this study. pEERM mainly contains various crucial regions including the flanking region of upstream *PsbA2* sequence, promoter sequence of *PsbA2* (P_psbA2_), multiple cloning sites of *Xba*I, *Pst*I and *Spe*I, chloramphenicol resistance cassette and the flanking region of downstream *PsbA2* sequence, respectively. Construction of a recombinant pEERM_*aas* plasmid (Table 1) firstly started by PCR amplifying the homologous *aas* gene fragment encoding AAS from *Synechocystis* sp. PCC 6803 genomic DNA template using a specific pair of primers, Aas_F and Aas_R (Table 2). The amplified *aas* fragment was then ligated into pEERM vector between the sites of *Xba*I and *Spe*I locating downstream of *PsbA2* promoter. For a recombinant pEERM_*LipA*/*AccDACB* (Table 1), the flanking region replacements in pEERM vector of both upstream and downstream *PsbA2* sequences were performed with the flanking regions of both upstream and downstream *lipA* gene sequences (encoding lipase A) obtained from PCR using two pairs of primers including USlipA_F and USlipA_R and DWlipA_F and DWlipA_R (Table 2), respectively. On the other hand, the inserted *accDACB* gene fragments encoding ACC were obtained by PCR (primer sequences shown in Table 2). All gene fragments were ligated with end-terminal sequence removing and sequentially cloned into pEERM plasmid between the *Xba*I and *Spe*I sites. Moreover, the recombinant pEERM_*Aar*/*Aas* plasmid (Table 1) was constructed by replacing both upstream and downstream regions in pEERM vector with both upstream and downstream regions of *aar* gene obtained by PCR using specific pairs of primers including USaar_F and USaar_R and DWaar_F and DWaar_R (Table 2), respectively.

**Table 2:** Primers used for PCR amplification, sequencing and determination of gene location

Name	Sequences
UPAar_F	5′-AGATCTAGGGACGGAACAAACCCTCCAAAGC-3′
UPAar_R1	5′-GAAGATCCTTTGATTTTGCCGACAGGATAGGGCGTGTGTGGA-3′
UPAar_R2	5′-GAATTCAAAAAAAGGATCTCAAGAAGATCCTTTGATTTTGCCGACAGGA-3′
DWAar_F	5′-GGATCCCATTGATAATAGTCAGAATAAATAG-3′
DWAar_R	5′-GTCGACCCTTTAGTAGCTCTTTAGGGGTTAA-3′
UPlipA_F	5′-TAGAGAAGATCT CAGGCCCTACGTCGTCATAATCCTG -3′
UPlipA_R1	5′-GAAGATCCTTTGATTTTGTGGATTGGAAAGGGATTAGTCTTC-3′
UPlipA_R2	5′-TAGAGAGAATTCAAAAAAAGGATCTCAAGAAGATCCTTTGATTTTGTGGATTGGA-3′
DWlipA_F	5′-TAGAGAGGATCCTAGGTTCTACAAACTCAGCAAACGG-3′
DWlipA_R	5′-TAGAGAGTCGACAGGTCAACCAAGATTCGGTGCACCA-3′
AccA_F	5′-TCTAGATAGTGGAGGTACTAGAATGAGTAAAAGTGAGCGTCGTG-3′
AccA_R	5′-CTGCAGCGGCCGCTACTAGTTTACACCGCCGTTTCTAAAAATTG-3′
AccB_F	5′-TCTAGATAGTGGAGGTACTAGAATGGACTACAAGGATGACGATGACAAG-3′
AccB_R	5′-CTGCAGCGGCCGCTACTAGTCTAGGGTTTAATCCACATTAGGG-3′
AccC_F	5′-TCTAGATAGTGGAGGTACTAGAATGCAATTCGCCAAAATTTTAATTGCC-3′
AccC_R	5′-CTGCAGCGGCCGCTACTAGTCTAGGGTGTTAAATGCTCTTCG-3′
AccD_F	5′-TCTAGATAGTGGAGGTACTAGAATGTCTCTATTTGATTGGTTTG-3′
AccD_R	5′-CTGCAGCGGCCGCTACTAGTTTAACCATCTTGATTGACGGAAA-3′
UUPPsbA2_SF	5′-GTGATGCCTGTCAGCAAAACAACTT-3′
Aas_F3	5′-AGACAATCTAGAGTGGACAGTGGCCAT-3′
Aas_R5	5′-GGAGATGGTTCAAGCTCAGG-3′
Aas_F4	5′-ACTCCCTAGAAAGAAGCGCC-3′
Aas_R6	5′-ATAAACACTAGTTTAAAACATTTCGTC-3′
Aas_SR	5′-GGCTATTCCAATGGATTTGAGGTTG-3′
Cm_SF	5′-GGCAGAATGCTTAATGAATTACAACAG-3′
Cm_SR	5′-CTGAAATGCCTCAAAATGTTCTTTACG-3′
UUPF_Aas	5′-GCGATCGCCGTCAATTTTCGATCAG-3′
pE_SF	5′-CATTACGCTGACTTGACGGG-3′
pE_SR	5′-AGGTATGTAGGCGGTGCTAC-3′
UUPF_LipA	5′-ACAGGGCCAGGTGGGAGAAATTTTG-3′
AccA_SR	5′-CTACCGGCCAATCAAGTTTGCAC-3′
UUPF_Aar	5′-CAAAAGTAATGAGGTCGTTTTACCC-3′
CUPAar_SF	5′-CTACCGGCCAATCAAGTTTGCAC-3′

### Natural transformation of recombinant plasmid into *Synechocystis* cells

*Synechocystis* wild-type cells, grown in 50 mL-BG_11_ medium for 2–3 days until reaching an OD_730_ of about 0.5, were harvested by centrifugation at 6000 rpm (4025×*g*). Obtained cell pellet was resuspended in 500 µL of new BG_11_ medium followed by the addition of 10 µg of each recombinant plasmid. The cell suspension was incubated at 28 °C for 6 h by inverting the mixture tube every 2 h before spreading on a 0.45 µm sterile nitrocellulose membrane placed over the normal BG_11_-agar plate. After 24 h incubation, the membrane was transferred onto BG_11_-agar containing 35 µg/mL of chloramphenicol. Normally, survived colonies were obtained within 3–4 weeks of incubation. Generated transformants were further examined for their gene location by PCR using selected, specific primers (Table 2).

### Determination of intracellular pigment content

Total chlorophyll *a* (Chl *a*) and carotenoid (Car) contents were extracted by *N,N*-dimethylformamide (DMF), and their contents were determined by measuring the absorbance at 461, 625 and 664 nm using a spectrophotometer [19, 20]. The Chl *a* and Car contents were normalized to a cell number corresponding to 1.0 × 10^8^ of the cells [19–21].

### Measurement of oxygen evolution

Harvested cells were incubated in the dark for 30 min before measuring their relative O_2_ evolution rate of cells under saturated white light illumination using Clark-type oxygen electrode (Hansatech instruments, UK) at 25 °C. The O_2_ evolution rate was represented as µmol/mg Chl *a*/h [22].

### Determinations of total lipid and unsaturated lipid contents

During cultivation, fifteen mL-cell cultures of either WT cells or engineered strains were harvested by centrifugation at 6000 rpm (4025×*g*), at room temperature for 10 min. Ten mL of a CHCL_3_:MeOH (3:1 ratio) mixture was added and incubated in a 55 °C water bath for 2 h. After that, ten mL of distilled water was added into the reaction tube and mixed. The sample mixtures were further incubated at room temperature for 10 min and separated by centrifugation at 6000 rpm (4025×*g*), room temperature for 10 min. The aqueous phase was discarded whereas the chloroform phase was collected for lipid determination. All lipids dissolved in chloroform were determined by acid–dichromate oxidation method [23]. One mL of dissolved lipid sample was added into 2 mL of concentrated sulfuric acid (H_2_SO_4_, 98%) and mixed vigorously using vortex. After that, 2 mL of 0.167 M potassium dichromate (K_2_CrO_7_) solution was added before boiling the mixture for 30 min. After the mixture was cooled down to room temperature, 2 mL of distilled water was added. The total lipid content was determined spectrophotometrically by measuring its absorbance at 600 nm. A commercial standard canola oil was prepared as control. The calculated content of total lipid was represented as % w/DCW.

The unsaturated lipid content was determined by a colorimetric sulfo-phosphovanillin (SPV) reaction method [24]. One mL of dissolved lipid was added into 2 mL of concentrated H_2_SO_4_ (98%), mixed and vigorously vortexed. Then, the mixture was boiled for 30 min and cooled down to room temperature. The 2 mL mixture of 17% H_3_PO_4_ and 0.2 mg/mL vanillin (1:1) was added into the solution and mixed. The total unsaturated lipid content was then determined by measuring absorbance of the reaction mixture at 540 nm using spectrophotometer. The commercial standard *γ*-linoleic acid (C18:3) was prepared in the same way as sample. The calculated content of total unsaturated lipid was represented as % w/DCW.

### Reverse transcription PCR

Total RNA was extracted from cells using TRIzol^®^ Reagent (Invitrogen) and treated with RNase-free DNaseI (Fermentas) to remove the genomic DNA contamination before converting to cDNA using SuperScript™ III First-Strand Synthesis Kit (Invitrogen). The obtained cDNA was used as a template in PCR of genes involved in lipid biosynthesis including *accA, aas, plsX, lipA* and *aar* using corresponding RT-PCR primers listed in Table 3. The PCR products were checked by 1% (w/v) agarose gel electrophoresis. Band intensity quantification was also performed using Syngene^®^ Gel Documentation (Syngene, Frederick, MD).

**Table 3:** Primer used for RT-PCR reactions

Target gene	Name	Primers	PCR product size (bp)
*16s*	16s_F	5′-AGTTCTGACGGTACCTGATGA-3′	521
	16s_R	5′-GTCAAGCCTTGGTAAGGTTAT-3′	
*plsX*	PlsX_F	5′-AAGGGGTGGTGGAAATGGAA-3′	488
	PlsX _R	5′-AAGTAGGTCCCTTCCTTCGG-3′	
*accA*	AccA_F	5′-ATGCACGGCGATCGAGGAGGT-3′	428
	AccA_R	5′-TGGAGTAGCCACGGTGTACAC-3′	
*aas*	Aas_F_RT	5′-CCCATTGAAGATGCCTGTTT-3′	304
	Aas_R _RT	5′-GTGCTGGGATAAAACGGAAA-3′	
*phaA*	PhaA_F	5′-TCAGCCGGATAGAATTGGACGAAGT-3′	432
	PhaA_R	5′-CAAACAAGTCAAAATCTGCCAGGGTT-3′	
*lipA*	LipA_F	5′-TTGGCGGAGCAAGTGAAGCAAT-3′	379
	LipA_R	5′-CATGGACCAGCACAGGCAAAAT-3′	
*aar*	Aar_F	5′-GGGAGATATTGGTAGCGCCG-3′	394
	Aar_R	5′-CCGCAAAACAGGCGAACATT-3′	

### Nile red staining

To investigate the presence of neutral lipids, the Nile red method [25] was used. One hundred µL of cell culture was stained with 30 µg/mL of Nile red solution containing 0.9% (w/v) NaCl and further incubated in the dark overnight. After that, the stained cells were smeared on the glass slide and visualized under the fluorescent microscope (Olympus DP72, USA).

### Analysis of fatty acid composition

For analysis of intracellular fatty acid composition, total lipids were extracted from 500 mL of cell culture with OD_730_ of about 0.5. The method was modified according to O’Fallon et al. [26] in order to generate fatty acid methyl esters (FAMEs). Mixture of methanol and 1 N KOH (1:3 ratio) was added to cell pellet and incubated in a 55 °C water bath for 1.5 h. Then, concentrated sulfuric acid (98%) was added and immediately mixed by inverting the tube. Equal volume of hexane was then added to the reaction tube and mixed with vortex. The hexane fraction was transferred to gas vials for GC–MS/MS detection. The data are shown as the percentage of fatty acid composition in *Synechocystis* cells.

## Results

**Fig. 2:**
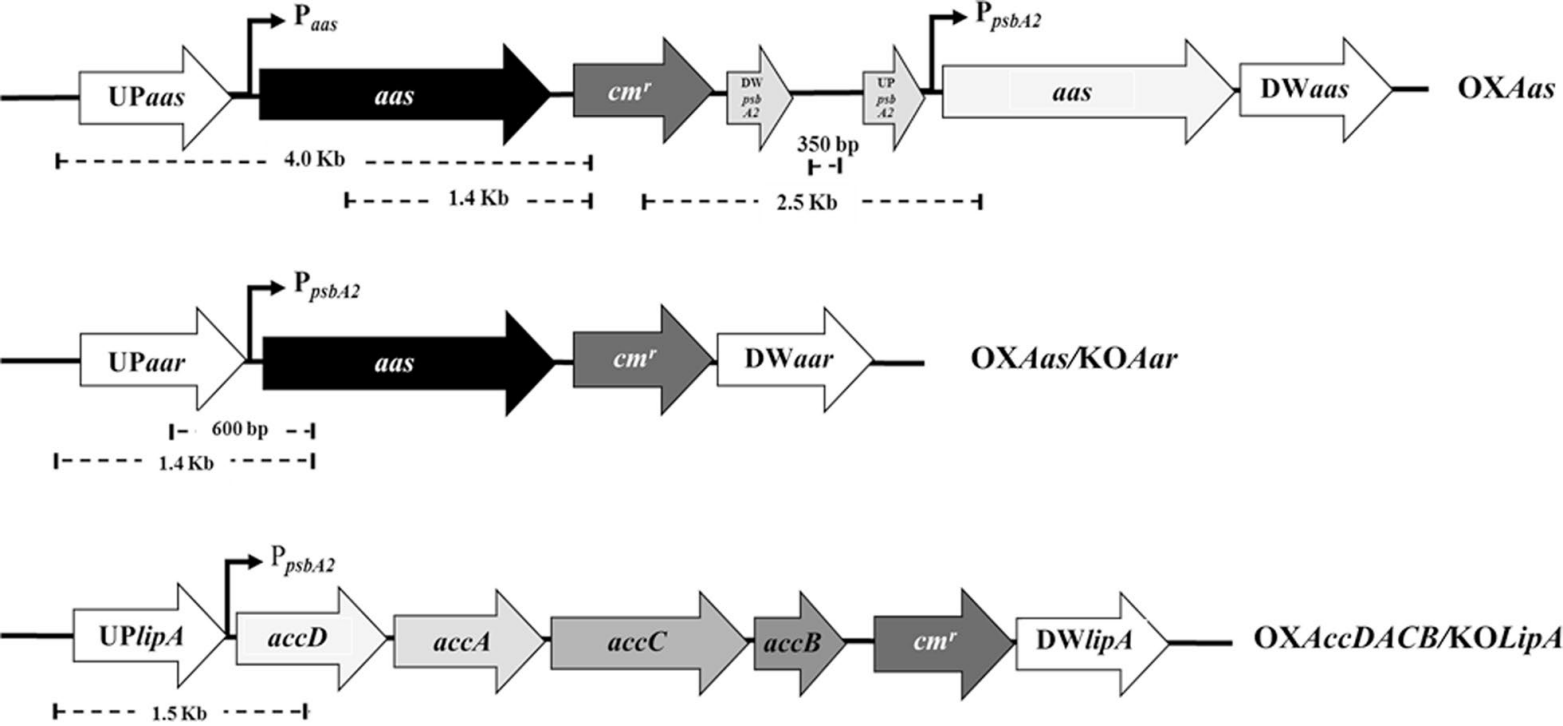
Outline maps representing gene locations in all *Synechocystis* engineered strains. OX*Aas* strain (upper) was singly recombined with *Aas* gene locus whereas OX*Aas****/***KO*Aar* strain (middle) was generated by interrupting *aar* gene with *aas* gene fragment insertion. Finally, OX*AccDACB*/KO*LipA* strain (bottom) was constructed by inserting a cassette fragment of *accD, accA, accC* and *accB* to disrupt *lipA* gene

**Fig. 3:**
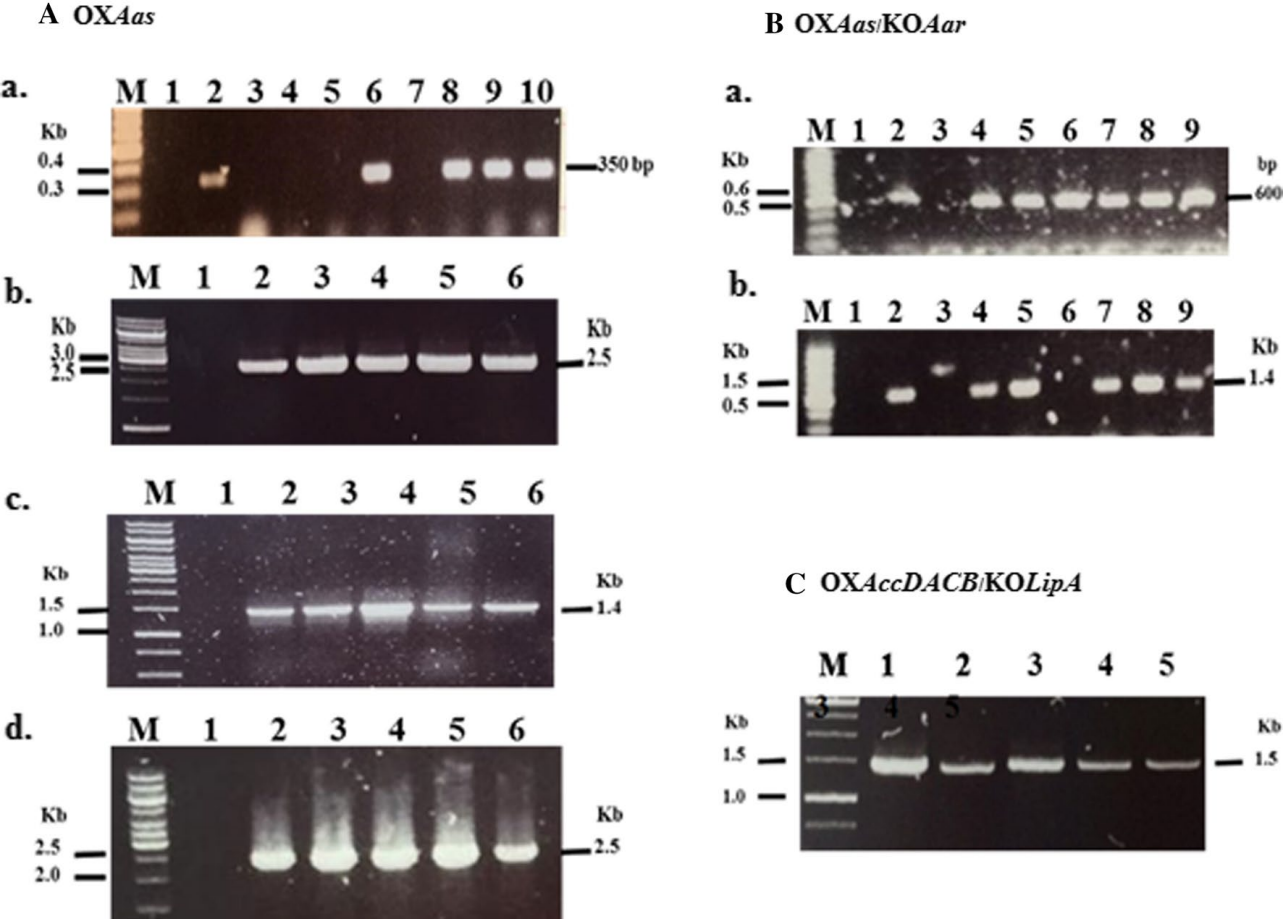
Confirmation of each gene location by PCR analysis using specific pairs of primers (Table 2) in each engineered strain including OX*Aas* (**A**), OX*Aas*/KO*Aar* (**B**) and OX*AccDACB*/KO*LipA* (**C**) strains in this study. The location of *aas* gene fragment in OX*Aas* was checked using a pair of pE_SF and pE_SR primers for pEERM core structure (a). Lane M: GeneRuler™ DNA ladder (Fermentas), lane 1: negative control using WT as template and lanes 2–10: clone numbers 1 to 9, For Cm_SF and Aas_SR (b) primer, lane M: GeneRuler™ DNA ladder (Fermentas), lane 1: negative control using WT as template and lanes 2–6: clone numbers 1 to 5. In (c), the pair of Aas_F6 and Cm_SR (c) primers was used, lane M: GeneRuler™ DNA ladder (Fermentas), lane 1: negative control using WT as template and lanes 2–6: clone numbers 1 to 5. The UUPSF_Aas and Cm_SR (d) primer, lane M: GeneRuler™ DNA ladder (Fermentas), lane 1: negative control using WT as template and lanes 2–6: clone numbers 1 to 5. Confirmation of gene location in OX*Aas*/KO*Aar* (**B**) using a pair of CAar_F and Aas_SR (a) primer, Lane M: GeneRuler™ DNA ladder (Fermentas), lane 1: negative control using WT as template and lanes 2–9; clone numbers 1 to 8 whereas UUPSF_Aar and Aas_SR (b) primers was used. Lane M: GeneRuler™ DNA ladder (Fermentas), lane 1: negative control using WT as template and lanes 2–11: clone numbers 1 to 10. The gene location in OX*AccDACB*/KO*LipA* (**C**) using pair of UUPSF_lipA and AccD_SR primer, Lane M: GeneRuler™ DNA ladder (Fermentas) and lanes 1–5; clone numbers 1 to 5

After the recombinant plasmids pEERM_*aas*, pEERM_*Aar/Aas* and pEERM_*LipA/AccDACB* (Table 1) were successfully constructed, they were separately transformed into *Synechocystis* WT cells generating the strains OX*Aas*, OX*Aas*/KO*Aar* and OX*AccDACB*/KO*LipA*, respectively. The obtained transformants grown on BG_11_ agar plate containing 35 µg/mL chloramphenicol were randomly selected and examined for their respective gene locations by PCR using various specific pairs of primers (Table 2). Obtained PCR products when using selected primers of each strain are shown in Fig. 3. The data revealed that the engineered strains OX*Aas*, OX*AccDACB/*KO*LipA* and OX*Aas/*KO*Aar* were successfully obtained. In Fig. 3A.a, the pEERM core structure was examined using primers pE_SF and pE_SR generating a DNA fragment of 350 bp (Table 2 and Fig. 2). WT (lane 1) contained no pEERM vector whereas the vector was observed in transformants or OX*Aas* strain (lanes 6, 8, 9 and 10). Interestingly, OX*Aas* possessed a single homologous recombination since a size of 2.5 kb between *cm*^*r*^ and *aas* locus was observed in OX*Aas* (lanes 2–6) except in WT (lane 1) (Fig. 3A.b). Additionally, 1.4 kb and 2.5 kb fragments were observed in *OXAas* except in WT (lane 1) by PCR using the two pairs of Aas_F6 and Cm_SR primers (Fig. 3A.c), and UUPSF_Aas and Cm_SR (Fig. 3A.d) primers, respectively (Table 2 and Fig. 2). An interruption of the *aar* gene by inserting *aas* gene fragment generated the OX*Aas/*KO*Aar* strain (Fig. 2). By PCR amplification using a pair of CAar_F and Aas_SR primers and another pair of UUPSF_Aar and Aas_SR primers (Table 2 and Fig. 2), 600 bp and 1.4 kb fragments were observed in strain OX*Aas/*KO*Aar* (Fig. 3B.a, b). For strain OX*AccDACB/*KO*LipA*, the native *lipA* gene was disrupted by a cassette fragment of *accD*, *accA*, *accC* and *accB* with homologous recombination using the flanking region of *lipA* gene (Fig. 2). A correct gene location was demonstrated for strain OX*AccDACB/*KO*LipA* (lanes 1–5) after being examined by PCR using a pair of UUPSF_lipA and AccD_SR primers (Fig. 3C).

**Fig. 4:**
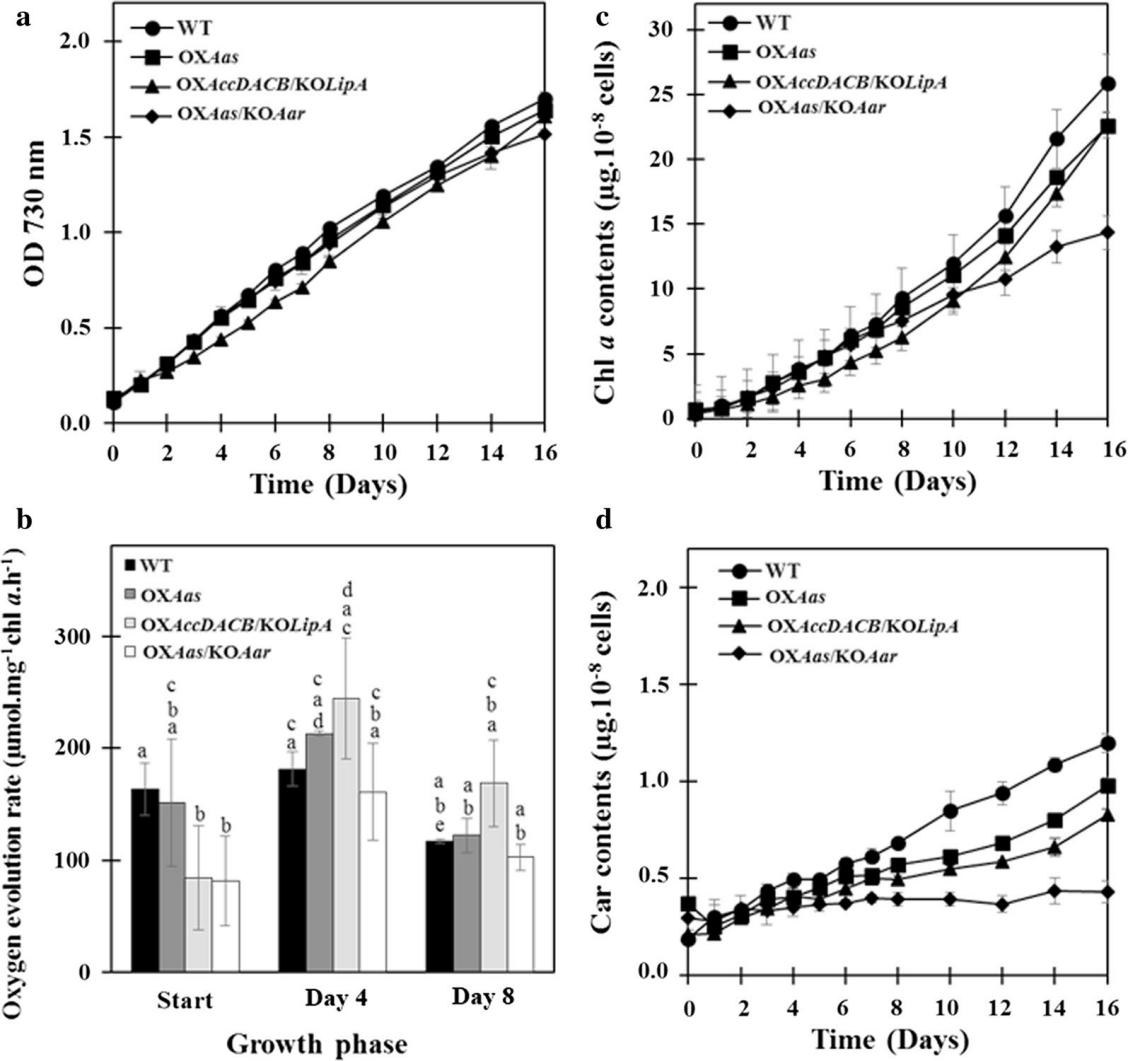
Growth curve (**a**), oxygen evolution rate (**b**), chlorophyll *a* content (**c**) and carotenoid content (**d**) of wild type, OX*Aas*, OX*AccDACB*/KO*LipA* and OX*Aas*/KO*Aar Synechocystis* strains grown in BG_11_ medium. The error bars represent standard deviations of means (mean ± SD, *n *= 3). Means with the same letter are not significantly different (in **b**) with the significance level at *P* < 0.05

Cell growth of WT and all engineered strains is shown in Fig. 4a. All engineered strains grew slightly slower than WT, in particular OX*AccDACB/*KO*LipA.* Oxygen evolution rates, representing the photosynthetic efficiency of the cells, were monitored in three growth stages including start, day 4 and day 8 of cultivation (Fig. 4b). WT cells gave a slight decrease of oxygen evolution rate at day 8 of growth whereas the oxygen evolution rates of all engineered strains showed no changes at both day 4 and day 8. The intracellular pigments including chlorophyll *a* and carotenoid contents during cultivation (Fig. 4c, d respectively) depicted the significant differences of WT and engineered strains, which were apparent during 8–16 days cultivation. Chlorophyll *a* and carotenoid contents of the engineered strains were significantly lower when compared to WT. Interestingly, the OX*Aas/*KO*Aar* strain showed a constant level of carotenoids throughout the cultivation period.

**Fig. 5:**
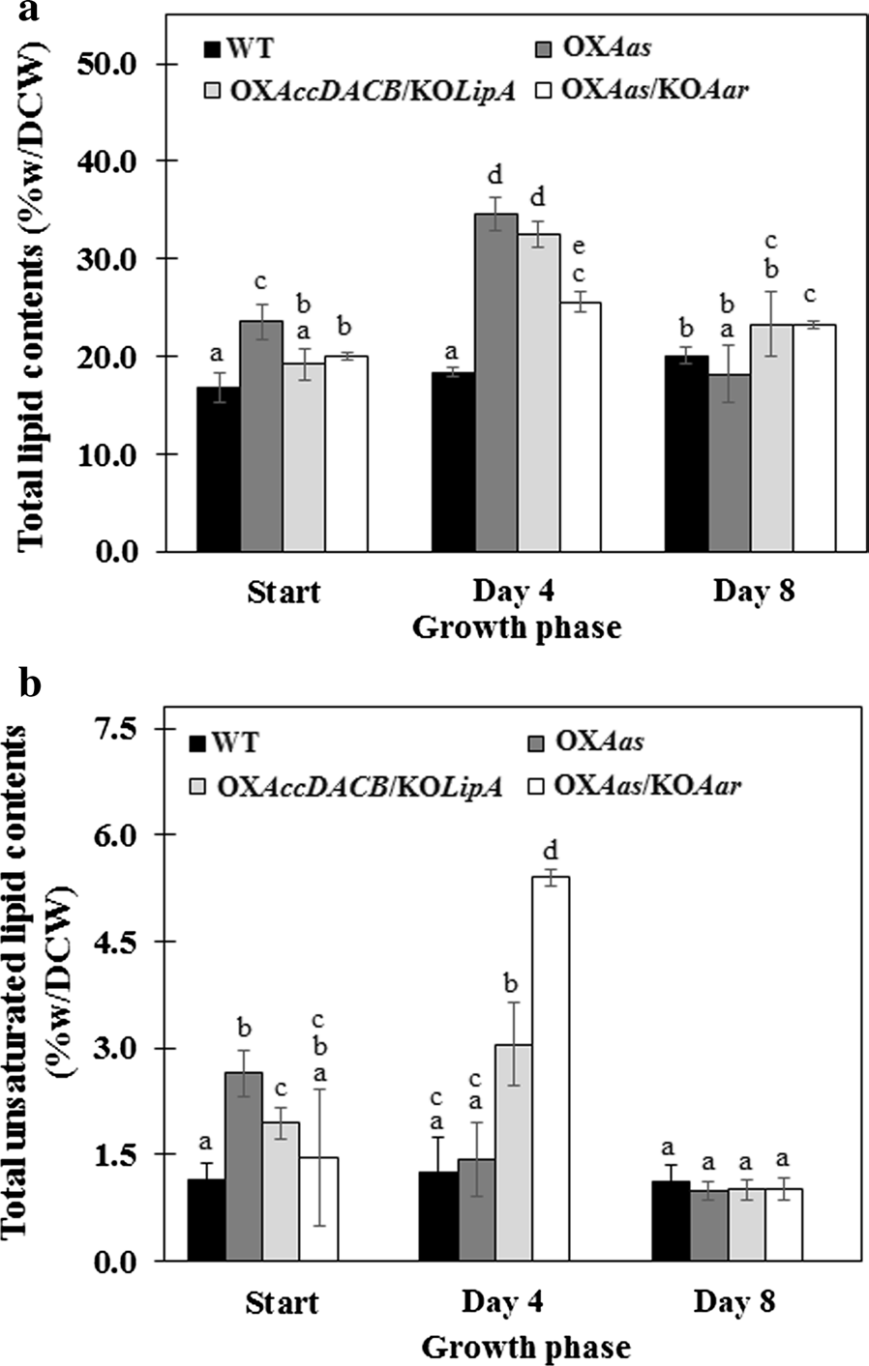
Total lipid content (**a**) and unsaturated lipid content (**b**) in wild type, OX*Aas*, OX*AccDACB*/KO*LipA* and OX*Aas*/KO*Aar Synechocystis* strains grown in BG_11_ medium. The error bars represent standard deviations of means (mean ± SD, *n *= 3). Means with the same letter are not significantly different with the significance level at *P* < 0.05

Total lipid contents in all strains are shown in Fig. 5a. At the start of cultivation, WT cells accumulated total lipids about 16.8% w/DCW and showed a slight increase at day 8 of cell growth. We noticed that at the start of cultivation the OX*Aas* produced the highest level of total lipids among all strains examined with about 23.5% w/DCW. Cells at day 4 increased the accumulation of total lipids in all engineered strains, especially OX*Aas* and OX*AccDACB/*KO*LipA* showing 34.5 and 32.5%w lipids/DCW, respectively. At day 8 of cultivation, the total lipid contents of both OX*Aas* and OX*AccDACB/*KO*LipA* decreased to similar level as that of WT. Additionally, although OX*Aas/*KO*Aar* did not induce a sharp increase of total lipid content, an increase of total lipid level was observed along all growth phases when compared to WT. This was substantiated by the highest production rate of lipids observed in OX*Aas* strain at day 4 of cultivation (Table 4). It should be noted that the lipid titer of OX*Aas* was increased at a slower rate compared with the other two engineered strains after 4 days. Total unsaturated lipid contents produced by all strains are shown in Fig. 5b. In our observation, the intracellular amount of total unsaturated lipid in WT was 14-fold lower than total lipids. Results revealed that all engineered strains had significantly increased a growth-dependent unsaturated lipid production. When compared with that of WT, OX*AccDACB/*KO*LipA* showed a 2.3-fold higher unsaturated lipid content at day 4 of growth whereas OX*Aas/*KO*Aar* gave the highest level of about unsaturated lipid 5.4% w/DCW (Fig. 5b). Additionally, there was a notable increase of saturated palmitic acid (C16:0) in the engineered strains, especially in OX*Aas* showing higher than 70% (Table 5) when compared to WT [27]. The unsaturated oleic acid (C18:1) was induced in OX*AccDACB*/KOLipA and OX*Aas/*KO*Aar*.

**Table 4:** Lipid titer and production rate in *Synechocystis* sp. PCC 6803 wild type, OX*Aas*, OX*AccDACB*/KO*LipA* and OX*Aas*/KO*Aar* strains grown in BG_11_ medium

Strains	Lipid titer (mg/L)			Production rate (mg/L/day)	
	Start	Day 4	Day 8	Day 4	Day 8
*Synechocystis* WT	13.46 ± 1.23^a^	87.94 ± 2.20^b^	168.25 ± 6.92^e^	21.98 ± 0.55^g^	21.03 ± 0.86^g^
OX*Aas*	14.13 ± 1.10^a^	165.71 ± 8.14^c^	171.96 ± 34.13^e^	41.43 ± 2.03^h^	21.49 ± 2.27^g^
OX*AccDACB*/KO*LipA*	14.01 ± 1.46^a^	126.03 ± 20.34^d^	198.41 ± 9.91^f,e^	31.51 ± 5.09^i^	24.80 ± 1.24^g,i^
OX*Aas*/KO*Aar*	13.37 ± 2.06^a^	95.24 ± 12.88^b,d^	175.66 ± 27.23^e,f^	23.81 ± 3.22^g,i^	21.96 ± 3.40^g,i^

The error represents standard deviations of means (mean ± SD, *n* = 3)

Means with the same letter are not significantly different with the significance level at *P* < 0.05

**Table 5:** Fatty acid composition (%) measured by GC–MS/MS instrument in *Synechocystis* sp. PCC 6803 wild type, OX*Aas*, OX*AccDACB*/KO*LipA* and OX*Aas*/KO*Aar* strains grown in BG_11_ medium for 4 days

Fatty acid composition (%)	*Synechocystis* WT [27]	OX*Aas* (this study)	OX*AccDACB*/KO*LipA* (this study)	OX*Aas*/KO*Aar* (this study)
Palmitic acid (16:0)	40%	72%	64%	69%
Palmitoleic acid (16:1)	2%	3%	nd	nd
Oleic acid (18:1)	3%	nd	20%	30%
Linoleic acid (18:2)	10%	nd	15%	nd
α-Linolenic acid (18:3)	12%	2%	nd	nd
Unidentified peak	33%	23%	< 1%	< 1%

*nd* nondetectable

**Fig. 6:**
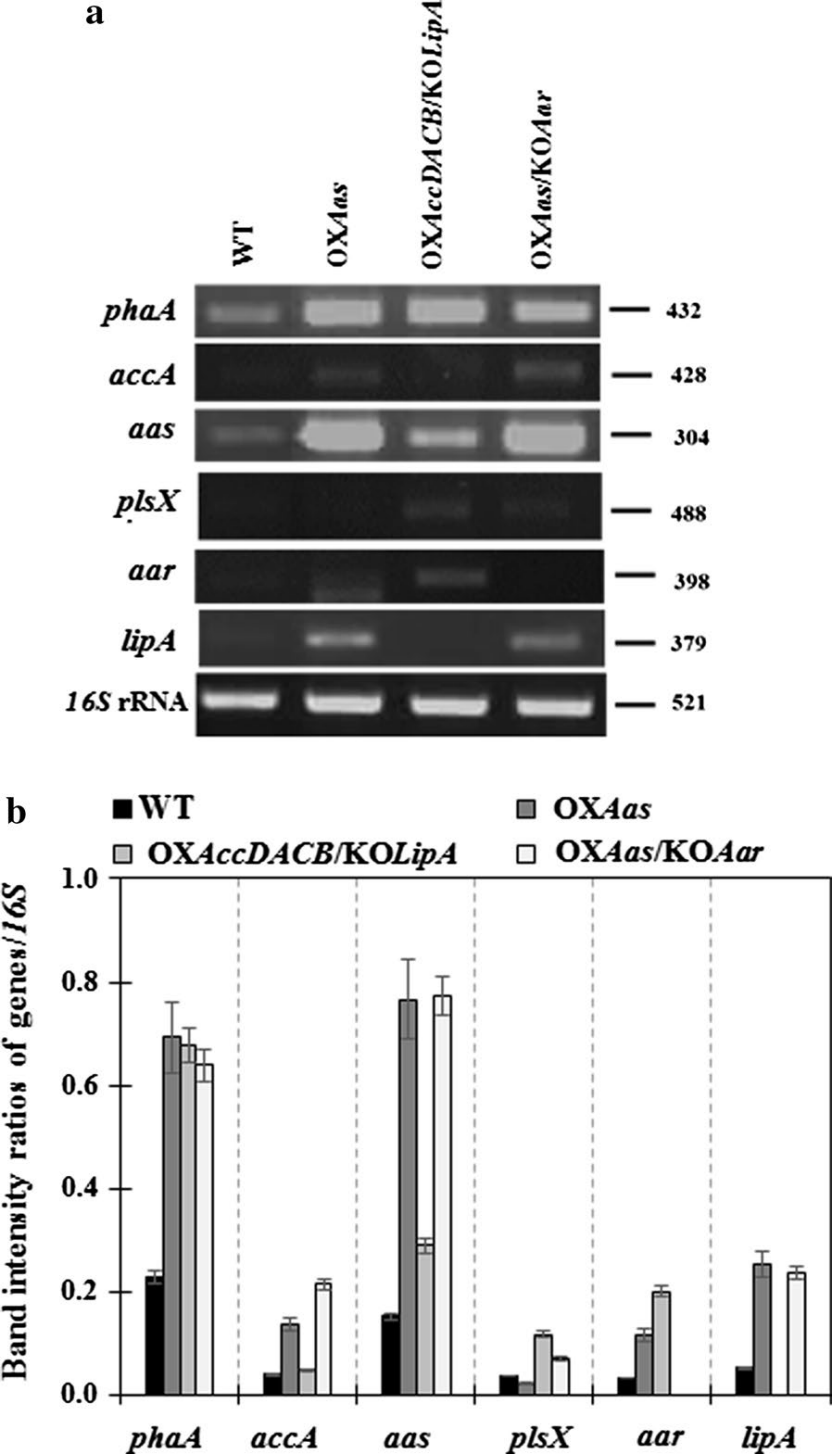
The transcript levels (**a**) of *pha A*, *accA*, *aas*, *plsX, aar, lipA* and *16S* rRNA genes of WT, OX*Aas*, OX*AccDACB*/KO*LipA* and OX*Aas*/KO*Aar Synechocystis* strains. The intensity ratios (**b**) of *phaA*/*16S* rRNA, *accA*/*16S* rRNA, *aas*/*16S* rRNA, *plsX*/*16S* rRNA, *aar*/*16S* rRNA and *lipA*/*16S* rRNA of all studied strains at log phase of cell growth analyzed by GelQuant.NET program

**Fig. 7:**
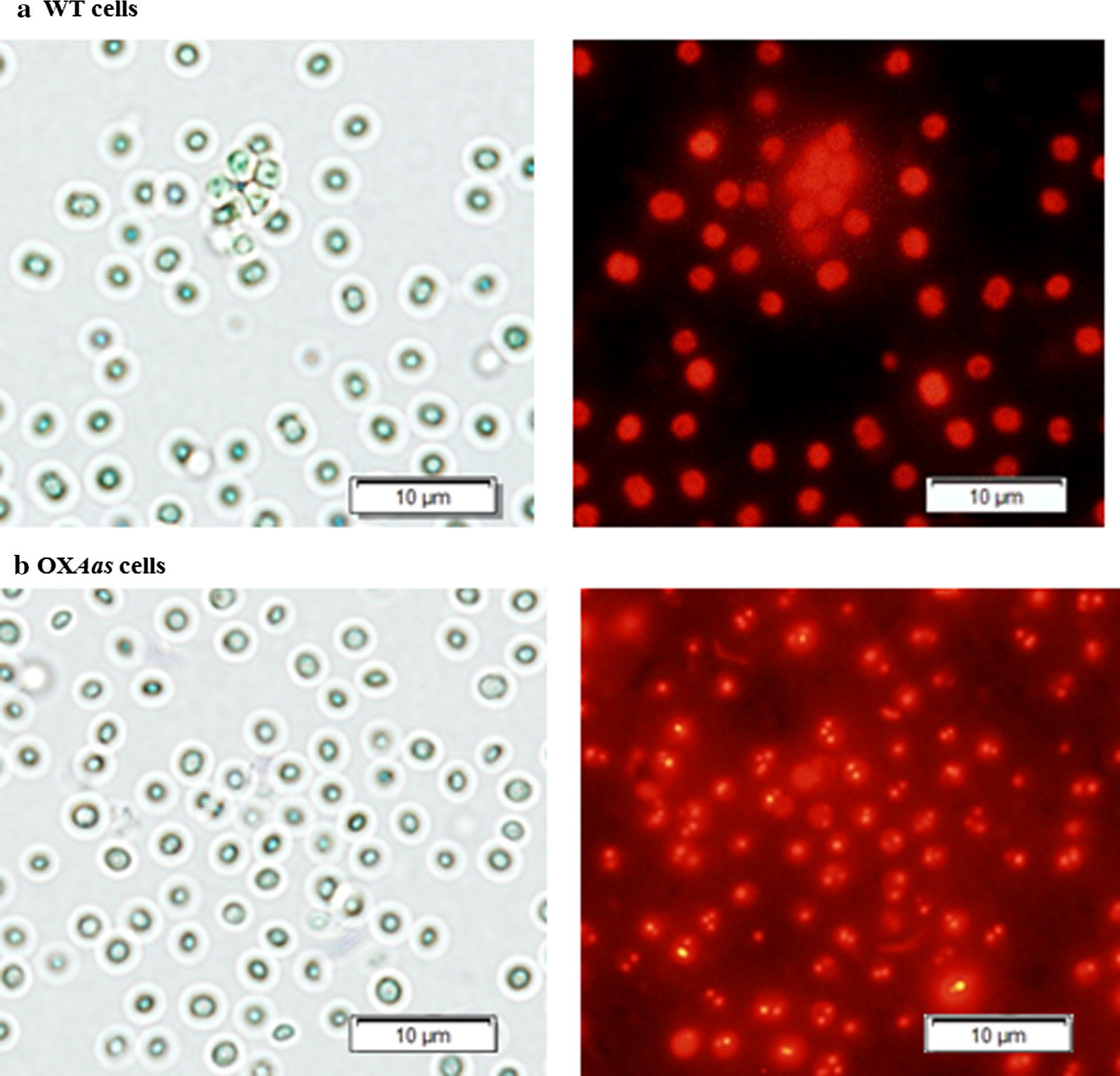
The Nile red staining of neutral lipids in *Synechocystis* sp. PCC 6803 wild type (**a**) and OX*Aas* (**b**) strain cells in BG_11_ medium at day 4 of cultivation. The stained cells were visualized under light and fluorescent microscopes with a magnification of ×100. It is noted that the focus setting in panel B with Nile Red staining was directed to PHB granules whereas the focus setting in panel A with Nile Red staining was directed to the whole cells

Results of gene expressions related to fatty acid biosynthesis and neighboring pathways (Fig. 1) under log growth phase of all strains, including *phaA*, *accA*, *aas*, *plsX*, *aar* and *lipA*, are shown in Fig. 6. The *aas* gene overexpression was confirmed with about a fivefold increase in both OX*Aas* and OX*Aas*/KO*Aar* compared to that in WT. In addition, a slight increase (about 1.2-fold) of *accA* transcript level was observed in OX*AccDACB*/KO*LipA*. Surprisingly, our results showed a distinct increase of *pha* gene expression, related to bioplastic PHB synthesis, in all engineered strains (Fig. 6). To check whether the engineered strains contained higher PHB than WT, we stained OX*Aas*, which showed the highest *phaA* transcript level, with Nile red and clearly observed significantly more PHB granules compared to those in WT cells (Fig. 7). On the other hand, the *aas* overexpression induced the *accA* transcript level, related to a gene of the multi-subunit acetyl-CoA carboxylase, in OX*Aas* and OX*Aas*/KO*Aar* (Fig. 6). For the *plsX*, related to phospholipid synthesis, the relative transcript levels increased in the engineered strains, especially in OX*AccDACB*/KO*LipA* and OX*Aas*/KO*Aar*. Moreover, both OX*Aas* and OX*AccDACB*/KO*LipA* showed higher relative transcripts levels of *aar*, related to alkane synthesis. Finally, an increased transcript level of *lipA* encoding the phospholipid hydrolyzing lipase was found in the strains OX*Aas* and OX*Aas*/KO*Aar*.

## Discussion

In this study, we constructed three engineered strains of the unicellular cyanobacterium *Synechocystos* PCC 6803: OX*Aas*/KO*Aar* and OX*AccDACB*/KO*LipA* segregated by double homologous recombination whereas the OX*Aas* was generated via single recombination (Figs. 2 and 3). The single integrative crossover or single recombination rarely occurs in *Synechocystis* PCC 6803 but may be more stable than a double recombination [28]. The genetic stability of the three engineered strains was likely to occur since the analysis of transcript levels in these strains was relatively unchanged during a period of over one year. We demonstrated that the metabolic engineering of all modified strains did not severely affect the cell growth except the intracellular pigment contents (Chl *a* and Car), in particular in strain OX*Aas*/KO*Aar* after 8–10 days of growth (Fig. 4c, d). However, the oxygen evolution rate, partly representing photosynthetic capacity and efficiency, of all strains studied was not significantly disturbed. On the basis of our empirical experiment and other reports, the normal range of oxygen evolution rate of *Synechocystis* PCC 6803 photoautotrophically cultivated was about 60–200 µmol/mg Chl *a/*h depending on strain and light intensity during cultivation [29], as well as if any stressful condition was applied [30]. In our study, the overexpression of *aas* with a simultaneous *aar* knockout showed the most significant reduction in OD_730_ as well as in intracellular pigments content. This reduction may partially correlate with the expression vector chosen and gene impact on cell metabolism. Coincidently, a previous study reported that a knockout of *aar* in *Synechocystis* caused not only a fourfold decline in growth when compared to *Synechocystis* WT cells but also a decreased oxygen evolution rate [31]. Due to the fact that the formation of alkane may partly modulate photosynthetic cyclic electron flow in cyanobacterial membranes, the disrupted *aar* gene may then cause a lowered growth and photosynthetic efficiency [32].

We also demonstrated that the day 4-growth phase of all strains was suitable for highest production of lipid metabolites. Our results indicate that the highest levels of lipids were observed in engineered strain OX*Aas* with about 34.5% w/DCW which is twofold higher than WT cells during log growth phase (Fig. 5). Sheng and co-workers previously reported that the intracellular lipid contents in cyanobacterium *Synechocystis* PCC 6803 were limited to a range between 10 and 15% w/DCW, significantly lower than that observed in the present study, with the majority being diacylglycerol components [33]. We observed an enhanced FFAs incorporation into fatty acyl-ACP, the initial precursor for lipid synthesis, resulting in significantly higher lipid level than WT (Fig. 1). In addition, an overexpression of the multi-subunit acetyl Co-A carboxylase gene (*accDACB*) in combination with a *lipA* knockout (strain OX*AccDACB*/KO*LipA*) resulted in a lipid content of about 32.5% w/DCW. The ACC encoded by a multi-subunit of *accA*, *accB*, *accC* and *accD* played a role as the rate-limiting step for the fatty acid biosynthesis [34, 35]. Coincidently, *accABCD* overexpressing *Escherichia coli* showed a sixfold increase of the fatty acid biosynthesis rate [8]. In our study, we designed not only an *accDACB* overexpression strain but also a strain with *accDACB* overexpression in combination with a *lipA* knockout (Fig. 1). This was done in order to prevent membrane lipids degradation to FFAs and potentially gain more lipids, as it has been shown that deleting *sll1969* (or *lipA*) encoding a putative lipolytic enzyme significantly decrease membrane lipid degradations [36]. On the other hand, the OX*Aas*/KO*Aar* strain with disrupted alkane production showed no increase of lipid production. Our results suggest that the lipid production in our engineered strains is partially associated with cell growth, in particular at day 4. Among engineered strains OX*Aas*/KO*Aar* showing a slightly lowered growth, a significant reduction of pigment contents and O_2_ evolution rate, a lower total lipid, had the highest total unsaturated lipids (Fig. 5b). In addition, the homeostasis of lipid balance might adjust the excess synthesized lipid down to normal level either via feedback inhibition of acetyl Co-A carboxylase by the fatty acyl-ACP or via lipid degradation. Additionally, the desaturation activity of the membrane lipids in *Synechocystis* has been located to the cytoplasmic and thylakoid membranes [37]. The increase of unsaturated lipid levels in OX*AccDACB*/KO*LipA* and OX*Aas*/KO*Aar* was noted which may be ascribed to the FA desaturation activity as supported by the decrease of palmitic acid (C16:0) as well as the increase of oleic acid (C18:1) composition when compared to that of OX*Aas* (Fig. 5b and Table 5). On the other hand, due to low molar C/N ratio of about 1/47 in BG_11_ medium, additional C in the form of acetate was shown to stimulate lipid production [27]. In this regard, the improvement of lipid synthesis in cyanobacteria is very challenging due to the small pool size of acetyl-CoA and the TCA fluxes [38]. Further improvements may redirect the upstream flux towards acetyl-CoA [39] or engineer the CO_2_-fixing machinery [40, 41].

We also examined relative gene expression detected by RT-PCR of genes related to the fatty acid biosynthesis and neighboring pathways (Figs. 1 and 6). One of the metabolic balance responses for lipid synthesis depends on feedback inhibition, herein fatty acyl-ACP which thereby inhibited back to ACC enzyme [11, 12]. Our results indicate that the *aas-*overexpressing strains (OX*Aas* and OX*Aas*/KO*Aar*) showed a significantly induced *accA* transcript level when compared to WT. In addition, the OX*Aas* strain showed an up-regulation of the *aar* transcript levels compared to WT. Interestingly, all OX strains contained significantly increased levels of *phaA* transcript related to bioplastic PHB synthesis. Furthermore, Nile-Red staining of strain OX*Aas* showed an increase in PHB granules compared to WT (Fig. 7). Thus, our observations may suggest that the overexpression of *acc* and *aas* influenced the acetyl Co-A synthesis enhancing both fatty acid synthesis and PHB production. Interestingly, in *Ralstonia eutropha* H16, a re-consumption of fatty acids is stimulated through the beta-oxidation pathway which iteratively removes two carbons from both fatty acid to yield acetyl-CoA, and from 3-hydroxyl-acyl-CoA, an intermediate in beta-oxidation, which enters the PHB synthetic pathway [42]. We also noted that increased levels of *lipA* transcripts were observed in the two strains OX*Aas* and OX*Aas*/KO*Aar* which needs more FFA substrate from phospholipid degradation. Our results are in agreement with a previous finding that *lipA* encoding lipase A catalyzes phospholipids hydrolysis [3] with a tight correlation with AAS which recycles the free fatty acids into fatty acyl-ACP. On the other hand, we propose that the increased levels of *plsX* transcript observed in strains OX*AccDACB*/KO*LipA* and KO*Aas*/KO*Aar*, compared to WT, and strain OX*Aas* are due to an influence of the *lipA* and *aar* knockouts, respectively. In *Streptococcus mutans*, the deletion of *PlsX* gene encoding an acyl-ACP:phosphate transcylase, evidently lost the central function of unsaturated fatty acid movement into membrane and the acid-adaptive response [43]. As expected, the transcript levels of *aar* in strains OX*Aas* and OX*AccDACB*/KO*LipA* were induced when compared to WT possibly caused by the *aas* overexpression resulting in an enhanced flux ability of the substrate fatty acyl-ACP.

## Conclusions

Our results of metabolic engineering of various genes involved in the fatty acid synthesis, phospholipid hydrolysis, alkane synthesis, and recycling of free fatty acid (FFA) in cyanobacterium *Synechocystis* sp. PCC 6803 indicated an increase in acetyl Co-A flux towards both routes of lipid and PHB syntheses as evident by their increased contents. Among the three engineered strains, OX*Aas* with enhanced recycling of FFA had the highest lipid content and lipid production rate after 4 days cultivation.

## Abbreviations

AAR: acyl-ACP reductase
AAS: acyl–acyl carrier protein synthetase
ACC: acetyl-CoA carboxylase
ACP: acyl carrier protein
Car: carotenoids
Chl *a*: chlorophyll *a*
CO_2_: carbon dioxide
DCW: dry cell weight
DMF: *N,N*-dimethylformamide
FAS: fatty acid synthase
FFA: free fatty acid
h: hour
m: meter
μg: microgram
mL: milliliter
min: minute
nm: nanometer
OD: optical density
PCR: polymerase chain reaction
PHB: polyhydroxybutyrate
rpm: revolutions per minute
s: seconds
SPV: sulfo-phosphovanillin
WT: wild type
